# Target temperature management in acute ischemic stroke

**DOI:** 10.3389/fmolb.2026.1728769

**Published:** 2026-01-15

**Authors:** Lan Gao, Ting Yang, Hong Chong, Longfei Wu, Jinming Han

**Affiliations:** Department of Neurology, Xuanwu Hospital Capital Medical University, Beijing, China

**Keywords:** acute ischemic stroke, application progress, stroke, targeted temperature management, treatment

## Abstract

Acute ischemic stroke (AIS) is an acute neurological deficit that results from focal cerebral ischemia associated with permanent brain infarction, and is a leading cause of death and disability worldwide. Considerable attention has been paid to reducing mortality and improving the prognosis of patients with AIS. Targeted temperature management (TTM), including hypothermia therapy, normothermia control, and febrile intervention, has been widely investigated in laboratory and preclinical studies and has provided substantial protection for neurological function. The effect of TTM on neurological function prognosis in patients with AIS has attracted significant attention. This review summarizes the related mechanisms of action, clinical applications, and short- and long-term effects of TTM on neurological function in AIS, providing a clinical reference for the application and prognosis of TTM in patients with AIS.

## 1 Introduction

Stroke is a leading cause of death and disability worldwide. For example, the estimated prevalence, incidence, and mortality rates of stroke in Chinese individuals aged ≥40 years have reached 2.6%, 500 per 100,000 person-years and 340 per 100,000 person-years respectively (Tu et al., 2023). Acute ischemic stroke is the primary cause of disability in adult stroke (Tsao et al., 2023). There are few effective treatments for AIS (Hurfor et al., 2020), but most therapeutic methods have failed to reduce mortality or improve neurological prognosis (Wassélius et al., 2022). With the aim of improving the neurological function and prognosis of AIS, the development of targeted temperature management (TTM) has attracted much attention. In 2011, this concept was proposed by five international professional associations to replace “therapeutic” or “mild hypothermia”, emphasizing the scope and importance of temperature management (Nunnally et al., 2011). Previous international expert consensus guidelines on the implementation of TTM in children (Topjian et al., 2019) and adults (Nolan et al., 2022; Chiu et al., 2021) with brain injury after cardiac arrest and severe neurological conditions have been released to exert protective effects on the nervous system. However, the clinical effects of TTM in AIS remain controversial owing to differences in research design and methods. In this review, TTM interventional strategies for patients with AIS, their clinical implementation, patient outcomes, and mechanisms are discussed. Furthermore, we propose a TTM research protocol for continuous improvement of TTM in clinical settings.

## 2 Literature search methodology

**FIGURE 1:**
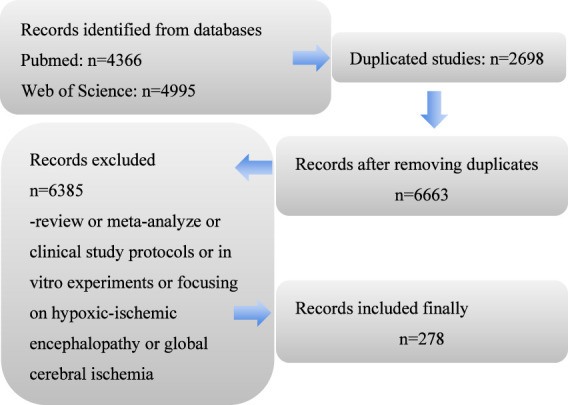
Study data. A total of 6,663 pre-clinical studies on PubMed and Web of Science were screened. After excluding the studies that did not meet the requirements, we conducted an in-depth analysis of 278 studies.

First, we conducted a systematic search of English-language literature indexed in the PubMed and Web of Science databases from 1990 to 2025. The search terms for the PubMed were: (Targeted Temperature Management OR therapeutic hypothermia OR induced hypothermia [Title/Abstract]) AND Acute Ischemic Stroke OR brain ischemia [Title/Abstract]) NOT hemorrhage [Title/Abstract] AND (English [Language]). The search terms for the Web of Science database were: TS = (brain OR cerebral OR intracranial OR cranial OR intracerebral) AND TS = (ischemic OR ischemia OR infarct OR infarction) AND TS = (hypothermia OR cooling OR lower temperature)) NOT TS = (hemorrhage OR hemorrhages OR intracranial hemorrhage OR intracranial hemorrhages OR cerebral hemorrhage OR cerebral hemorrhages OR bleeding) AND LANGUAGE: (English). These search strategies identified a total of 4,366 articles from PubMed and 4,995 articles from Web of Science. After removing 2,698 duplicate records across both databases, 6,663 unique studies remained for screening. Exclusion criteria encompassed review articles or meta-analyses, clinical study protocols, *in vitro* experiments, as well as studies focusing on hypoxic-ischemic encephalopathy or global cerebral ischemia. Ultimately, 22 articles were selected from 278 eligible publications for in-depth analysis and discussion. The literature screening criteria and process were summarized in Figure 1.

## 3 Mechanism of TTM in patients with AIS

### 3.1 Core mechanisms: Reducing cerebral metabolism and maintaining energy homeostasis

Therapeutic hypothermia is the primary method for implementing targeted temperature management in patients with AIS. The neuroprotective effect of hypothermia is fundamentally based on its ability to lower the cerebral metabolic rate. For every 1 °C decrease in body temperature, the cerebral metabolic rate is reduced by 6%–10%, thereby decreasing energy expenditure and providing critical time for the survival of ischemic brain tissue (Jo, 2022). Hypothermia maintains intracellular homeostasis through multiple mechanisms, particularly by preserving high-energy phosphate compounds, such as ATP and stabilizing tissue pH, thereby delaying the cascade of cell death triggered by energy depletion. Under ischemic conditions, the depletion of ATP leads to the dysfunction of the Na^+^/K^+^-ATPase pump, resulting in potassium efflux and massive influx of sodium and calcium ions. Intracellular calcium overload is a pivotal event that induces excitotoxicity-primarily mediated by glutamate-and activates various degradative enzymes, ultimately leading to neuronal death. Hypothermia effectively suppresses ischemia-induced excessive glutamate release and reduces calcium influx, mitigating excitotoxic injury (Kurisu and Yenari, 2018).

### 3.2 Acts on key molecular pathways: Anti-apoptotic, anti-inflammatory and antioxidant effects

#### 3.2.1 Inhibition of apoptotic pathways

A core effect of hypothermia is the stabilization of mitochondrial function. Within the intrinsic (mitochondrial) pathway, hypothermia upregulates the expression of the anti-apoptotic protein BCL-2 and suppresses the activation and translocation of pro-apoptotic proteins such as BAX. This stabilizes the mitochondrial membrane potential, reduces cytochrome C release, and consequently inhibits the cascade activation of caspase-9 and caspase-3, thereby blocking the apoptotic program. Hypothermia also exerts an inhibitory effect on the extrinsic apoptotic pathway. In the extrinsic (death receptor) pathway, hypothermia downregulates the expression levels of cell surface death receptors (e.g., Fas and TNF-R1) and their corresponding ligands (e.g., FasL). This downregulation diminishes the formation of the death-inducing signaling complex (DISC), subsequently inhibiting the activation of caspase-8 (Xiong et al., 2011). Furthermore, recent studies in a rat model of middle cerebral artery occlusion (MCAO) have demonstrated that hypothermic therapy may confer neuroprotection via a novel mechanism involving the regulation of miR-291b expression (Zhao et al., 2021), playing a crucial role in the context of mild hypothermia treatment for cerebral ischemia/reperfusion injury.

#### 3.2.2 Alleviation of inflammatory response

Post-ischemic inflammation is a pivotal factor in secondary brain injury. Hypothermia exerts potent anti-inflammatory effects. In animal stroke models, such as MCAO or intracerebral hemorrhage (ICH), therapeutic hypothermia has been shown to significantly suppress the over-activation of microglia and astrocytes, reduce the release of pro-inflammatory cytokines such as tumor necrosis factor-α (TNF-α), interleukin-1β (IL-1β) and IL-6, and inhibit the expression of intercellular adhesion molecule-1 (ICAM-1) on cerebrovascular endothelial cells. These effects effectively attenuate neutrophil infiltration into the ischemic brain tissue, thereby mitigating inflammation-induced secondary neuronal damage (Jin et al., 2016). Furthermore, hypothermia can inhibit the activation of key pro-inflammatory transcription factors, notably nuclear factor-kappa B (NF-κB). This inhibition prevents the nuclear translocation of NF-κB, thereby suppressing or down regulating the expression of pro-inflammatory and pro-apoptotic genes at the transcriptional level (Al-Ward et al., 2025).

#### 3.2.3 Mitigation of oxidative stress

During ischemia-reperfusion, mitochondrial dysfunction and aberrant enzymatic reactions lead to excessive production of reactive oxygen species (ROS) and reactive nitrogen species (RNS), overwhelming the scavenging capacity of the endogenous antioxidant system and triggering lipid peroxidation, protein denaturation, and DNA damage. Hypothermic therapy reduces the generation of free radicals and upregulates the endogenous anti-oxidant enzyme system, enhancing the activity of enzymes such as superoxide dismutase (SOD), glutathione peroxidase (GSH-Px), and reductases like glutathione (Chen et al., 2025), and then alleviating oxidative damage products (e.g., MDA, 8-OHdG) and preserving the integrity of biomacromolecules. It is important to note that antioxidant effects of hypothermia are not isolated. They are intricately intertwined with its anti-apoptotic and anti-inflammatory mechanisms. This multi-target, network-based mode of action provides a robust theoretical foundation for its clinical application.

### 3.3 Facilitation of neural repair and remodeling

**FIGURE 2:**
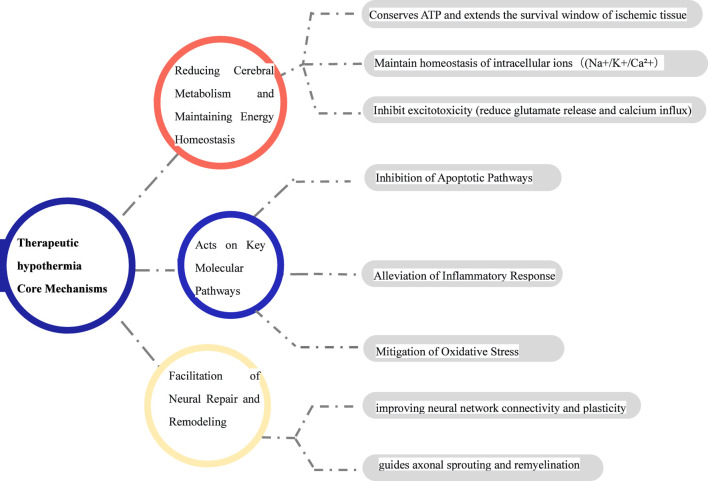
The neuroprotective effects of TTM (Targeted Temperature Management) are deeply rooted in its precise regulation of multiple cell death and injury pathways.

Research indicates that hypothermic therapy can significantly upregulate the expression of brain-derived neurotrophic factor (BDNF) and nerve growth factor (NGF) (Chen et al., 2025). These neurotrophic factors, by binding to their specific tyrosine kinase receptors, activate multiple downstream intracellular signaling pathways, including PI3K/Akt, MAPK/ERK, and PLCγ. The increased levels of BDNF not only directly inhibit neuronal apoptosis but also enhance presynaptic neurotransmitter release, promote postsynaptic glutamate receptor membrane trafficking, thereby consolidating long-term potentiation and improving neural network connectivity and plasticity. NGF primarily supports the survival and function of basal forebrain cholinergic neurons, playing a crucial role in the recovery of cognitive function. Furthermore, the synergistic upregulation of BDNF and NGF promotes the differentiation of neural stem/progenitor cells into neurons within the peri-ischemic region and guides axonal sprouting and remyelination. This lays the molecular groundwork for structural neural remodeling and functional compensation following stroke. This mechanism reveals that the benefits of hypothermic therapy extend beyond acute neuroprotection, exerting profound and positive effects on long-term neural repair and functional recovery. The core mechanisms of therapeutic hypothermia are summarized in Figure 2.

## 4 Application of TTM in patients with AIS

### 4.1 Initiation time of TTM

A comprehensive literature search was conducted, we summarized the clinical studies on TTM in AIS patients, and there was considerable variation in the initiation time of TTM in patients with AIS. Theoretically, TTM should be started as early as possible to prevent the deterioration of neurological function. With the increasing prevalence of intravenous thrombolysis and arterial thrombectomy techniques, the implementation of TTM after vessel reperfusion has become a trend. The most common implementation of TTM was within 6 h of stroke. For patients with AIS who received intravenous thrombolysis, interventions including ice-cold saline infusion during thrombolysis (Kollmar et al., 2009), mild hypothermia and local hypothermia devices (HDB-02) using semiconductor cooling technology (Bi et al., 2011), and head cooling with an SDL-V dual-control cranial cooling device have been previously used (Liu et al., 2019). Intravenous cooling with ice-cold saline through a Celsius catheter can be initiated quickly after thrombolysis (Lyden et al., 2016), whereas some studies were conducted within 30–180 min of the completion of intravenous thrombolysis (Lyden et al., 2005; Guluma et al., 2006). Patients with AIS who undergo thrombectomy typically receive systemic or selective endovascular cooling after vessel (Thomas et al., 2010; Bart van der Worp et al., 2019; Horn et al., 2014; Ji et al., 2014; Chen et al., 2016; Wu et al., 2018; Yue et al., 2023; Cheng et al., 2023; Bai et al., 2024). In addition, TTM was used within 12 h poststroke onset (Kammersgaard et al., 2000), as the temperature during admission is an independent predictor of prognosis during the first 12 h after stroke (Jørgensen et al., 1996). However, other studies (Georgiadis et al., 2001) have suggested that the time from symptom onset to the initiation can be as long as 58 h.

### 4.2 Implementation method of TTM

With the advancement of temperature regulation equipment and technology, TTM can be implemented using a surface cooling technique with a temperature feedback control system or an endovascular cooling technique, providing high-quality TTM for patients with AIS. Surface cooling technology has the advantages of being non-invasive and low-cost (Kammersgaard et al., 2000; Krieger et al., 2001; Schwab et al., 2001; Zhang et al., 2021), utilizing an efficient water circulation cooling system to regulate a patient’s local or whole-body temperature. Meanwhile, an alcohol bath and ice pack can be used to induce cooling. However, surface cooling techniques may lead to side effects such as shivering, imprecise temperature control, delays in reaching the target temperature, and rebound hyperthermia, particularly in intubated and pharmacologically sedated patients (Den Hertog et al., 2009).

For systemic endovascular cooling, a central venous heat exchange catheter is placed through the femoral vein and advanced into the inferior vena cava (Lyden et al., 2016; Lyden et al., 2005; Guluma et al., 2006; Thomas et al., 2010; Horn et al., 2014; Bai et al., 2024; Martin-Schild et al., 2009; Su et al., 2016), which is connected to an external mobile temperature management device to control the patient’s core temperature. This method is characterized by rapid induction and strict temperature control, which improve patient discomfort. This invasive method has potential risks including bleeding and thrombosis (Müller et al., 2014). Furthermore, local infusion of ice-cold saline seems to achieve the purpose of TTM, such as selective intra-arterial hypothermia, based on microcatheter technology. For patients undergoing mechanical thrombectomy, 4 °C ice normal saline is injected through the microcatheter after vascular recanalization, which can inhibit the transformation of ischemic penumaria, remove oxygen free radicals, and reduce inflammatory damage after acute infarction and ischemia reperfusion (Yue et al., 2023).

Another promising approach is drug-induced hypothermia: transient receptor potential cation channel 1 activators, neurotensin, opioids, endoccannabinoids, thyroxine derivatives, dopamine receptor agonists, gaseous hypothermia (xenon, hydrogen sulfide) and adenosine/adenine nucleotides (Ma et al., 2017; Han et al., 2021). The mechanism of action of drug-induced hypothermia affects the thermoregulatory center of the hypothalamus. The synergistic potential of hypothermic induction and neuroprotective effects has been demonstrated in preclinical studies. For example, chlorpromazine and prochlorperazine can safely and effectively induce hypothermia during ischemic periods, suggesting neuroprotective effects in both transient and permanent ischemia models (Geng et al., 2017). Hypothermia induced by dihydrocapsaicin (DHC) and intra-arterial regional cooling injection of reperfusion drugs can effectively improve acute ischemic injury (Wu et al., 2019). Nasal cooling may also serve as a TTM approach (Bardutzky et al., 2024; Poli et al., 2014), while further research is needed.

### 4.3 Combination therapy of TTM

When TTM begins, the patient’s consciousness is mainly divided into two states: awake and consciousness disturbance, which are affected by the disease itself, therapeutic methods, and target temperature regulation methods. In previous studies, the temperature regulation range of TTM was between 28 °C and 35.5 °C. In the awake state, patients can clearly perceive the discomfort caused by local or systemic temperature regulation. These discomfort symptoms are often managed with intravenous meperidine combined with oral droperidol or other medications (Kollmar et al., 2009; Lyden et al., 2016; Lyden et al., 2005; Guluma et al., 2006; Thomas et al., 2010; Bart van der Worp et al., 2019; Horn et al., 2014; Den Hertog et al., 2009; Martin-Schild et al., 2009). Gastric tubes can be used in patients who are unable to take oral medications, a gastric tube can be used (Bart van der Worp et al., 2019). The patient’s shivering threshold can be decreased to 33.4 °C using a combination of these drugs, while rarely causing sedation or respiratory adverse reactions (Wu et al., 2020). This method serves as the standard method for controlling shivering in most clinical trials. Magnesium sulfate has anti-convulsant effects, it is also seen in studies (Horn et al., 2014; Martin-Schild et al., 2009). For patients with AIS under general anesthesia, sedation and analgesia are achieved using a combination of midazolam, fentanyl, or dexmedetomidine. If necessary, neuromuscular blocking agents, (atracurium or vecuronium) can be used to induce nerve blockade (Ji et al., 2014; Georgiadis et al., 2001; Krieger et al., 2001; Schwab et al., 2001; Su et al., 2016; Hee Choi et al., 2021), preventing the stimuli caused by mechanical ventilation, temperature regulation and other procedures.

### 4.4 Monitor index of TTM

Clinical practice, hypothermia should be maintained for 12–24 h, with a target temperature being 33 °C–34 °C and slow rewarming over 12 h in clinical practice (Wu et al., 2020). During this period, precise temperature control is a complex yet crucial component as it has a significant impact on the effectiveness of TTM and patient recovery. The most commonly used method for accurate temperature monitoring is the bladder probe, whereas other sites, including the esophagus, tympanic membrane, and rectum, have been used in previous studies. The esophageal site is considered the preferred choice because it is close to the heart and lungs, providing a more accurate reflection of the core temperature (Mayer et al., 2004). Clinically, the monitoring site should be chosen based on the patient’s clinical condition and the medical environment. Furthermore, monitoring of laboratory parameters is required during the implementation of TTM, such as blood pH, electrolyte levels, coagulation and infection markers, and heart, liver, and kidney function indicators, to detect or prevent systemic complications. Comorbidities (hypertension, diabetes, hyperlipidemia, smoking, and atrial fibrillation) may affect therapeutic decisions and outcomes of AIS. Therefore, healthcare providers should closely monitor changes in patients’ conditions and promptly implement appropriate symptomatic treatments.

## 5 The impact of TTM on AIS patients

### 5.1 The impact of TTM on short-term neurological function

The National Institutes of Health Stroke Scale (NIHSS) is used to assess multiple aspects of neurological function, including level of consciousness, eye movement, facial expression, limb movement and language ability. The NIHSS is currently widely employed to evaluate the degree of neurological deficit in patients with AIS. In a preliminary pilot study, Kollmar et al. (2009) reported significant improvement in NIHSS scores using peripheral intravenous infusion of 4 °C ice-cold saline (25 mL/kg) combined with buspirone/meperidine for shivering prevention and management. However, this study had a small sample size (n = 20) and relatively low baseline NIHSS scores (median 5.5), which limits the generalizability of its findings. Bi et al. (2011) combined local mild hypothermia (using semiconductor technology) with intravenous thrombolysis and reported improvement of 24-h NIHSS scores. Nonetheless, their study indicated that this approach provided no additional neurological benefit compared to intravenous rtPA alone. In contrast, the randomized controlled trial by Thomas et al. (2010) employed systemic endovascular cooling combined with intravenous thrombolysis, and found no statistically significant advantage in NIHSS scores for the hypothermia group compared to the control group during both short-term (within 24 h) and long-term (1–3 months) follow-up periods. A detailed summary was provided in Table 1.

**TABLE 1:** Summary of key clinical trials on targeted temperature management in acute ischemic stroke.

First author	Sample size	NIHSS scores	Treatment methods	Intervention	Outcome	Conclusion
Kollmar et al. (2009)	20	5.5 (range 4–12)	Intravenous thrombolysis	General cold 0.9% saline (4 °C) was infused	NIHSS score was improved compared to the admission	Effective
Bi et al. (2011)	124	11.4 ± 2.8 (range 5–12)	Intravenous thrombolysis	Local hypothermia	NIHSS score (24h) was improved significantly	No benefit compared to IV rtPA alone
Thomas et al. (2010)	58	≥7	Intravenous thrombolysis	General intravascular cooling	Decreased NIHSS score between 1 and 3 months	Definitive efficacy trial is necessary to evaluate
Bart van der Worp et al. (2019)	98	11 (range 7–17)	Intravenous thrombolysis	Surface or endovascular technique	no difference between the groups on the mRS at 91 days	Hypothermia is unlikely to be a widely applicable
Horn et al. (2014)	75	17 (range15–18)	Intravenous thrombolysis or intra-arterial treatmen	Endovascular cooling catheter or surface cooling device	mRS at 3 months was improved significantly	Improved clinical outcomes
Wu et al. (2018)	113	17 (range 13–21)	Intra-arterial treatment or/and intravenous thrombolysis	Intra-arterial selective cooling infusion	mRS score was improved after 90 days, but was not statistically significant	IA-SCI with MT was safe, reduces infarct volume but does not improve clinical outcomes
Yue et al. (2023)	142	15 ± 7	Intra-arterial treatment	Intraarterial selective cooling infusion	mRS score was improved after 90 days	Selective intraarterial hypothermia combined with mechanical thrombectomy is safe and effective
Bai et al. (2024)	44	19 (range 16–20)	Endovascular thrombectomy	General systemic cooling	mRS score was improved after 90 days	Future randomized clinical trials are warranted to validate its efficacy
Kammersgaard et al. (2000)	73	NA	NA	General surface cooling with the “forced air”	SSS score was improved at 3 months	Future randomized clinical trials are warranted to validate its efficacy
Piironen et al. (2014)	36	11 (range 8–17)	Intravenous thrombolysis	General surface cooling	mRS score was improved after 90 days	Safe and feasible, despite the adverse events

These contradictory outcomes in short-term neurological function assessments likely stem from critical differences in study design. First, the timing of intervention varied: studies by Kollmar and Bi initiated early cooling (during thrombolysis or within 6 h of onset), whereas the intervention window in some trials (Thomas et al.) may have been later or more heterogeneous. Second, cooling methods and intensity differed: pilot studies may have employed relatively aggressive rapid cooling (e.g., high-volume ice-cold saline infusion), whereas large-scale RCTs using standardized, more controlled endovascular or local hypothermia techniques might yield different physiological effects. Furthermore, differences in patient populations are crucial; factors such as baseline stroke severity (NIHSS score), infarct location (anterior VS. posterior circulation), and endovascular therapy can significantly influence the response to hypothermia treatment. For instance, in patients with large vessel occlusion and higher NIHSS scores undergoing mechanical thrombectomy, studies by Wu et al. (2018) and Yue et al. (2023) suggest that immediate selective intra-arterial cold saline infusion via microcatheter (a form of regional and high-intensity cooling) after thrombectomy may more effectively mitigate reperfusion injury, leading to observed trends of improved short-term neurological scores. In summary, these discrepancies in short-term neurological outcomes are not coincidental, but rather reflect the profound impact of the complex interplay among therapeutic time window, technical approach, and patient characteristics on the efficacy of TTM as a multifaceted intervention.

### 5.2 The impact of TTM on long-term neurological function

The modified Rankin Scale (mRS) is routinely employed to assess long-term functional outcomes at 3-month post-stroke. Current evidence indicates that the impact of TTM on long-term prognosis is inconsistent and closely associated with the specific intervention strategy employed. For the strategy combining intravenous thrombolysis with systemic hypothermia, multiple high-quality randomized controlled trials (RCTs) as shown in Table 1, have yielded relatively consistent negative results. For instance, studies by Thomas et al. (2010), Bart van der Worp et al. (2019), and Piironen et al. (2014) failed to demonstrate that the addition of systemic hypothermia to standard intravenous thrombolysis significantly increased the proportion of patients achieving favorable functional outcomes (mRS 0-1 or 0–2) at 3 months. Notably, the trial by Bart van der Worp et al. (2019) was even prematurely terminated due to inadequate trial center experience and insufficient study design. Potential explanations for these negative findings include: 1) Delayed initiation of systemic hypothermia, which may have missed the optimal therapeutic window for neuroprotection; 2) Higher rates of complications, particularly infections (e.g., pneumonia) and cardiovascular events (e.g., bradycardia), which themselves could counteract the potential benefits of hypothermia; and 3) The relatively modest intensity and duration of cooling within the standardized protocols of large-scale clinical trials may be insufficient to elicit a significant clinical outcome.

In contrast, mechanical thrombectomy combined with regional intra-arterial hypothermia demonstrates potential for altering the disease trajectory, although the results require cautious interpretation. The RCT by Yue et al. (2023) found that for patients with anterior circulation large vessel occlusion, immediate selective intra-arterial cold saline infusion following mechanical thrombectomy significantly increased the rate of favorable functional outcomes (mRS 0–2) at 3 months. However, a similar study by Wu et al. (2018) did not achieve statistical significance. This inconsistency highlights that the technique remains investigational, and its efficacy may be finely modulated by critical variables including: patient selection (stricter imaging criteria to define a salvageable ischemic penumbra), the “dose” of cooling (encompassing the temperature, flow rate, total volume and duration of cold saline infusion), and precise timing relative to reperfusion. Furthermore, regional intra-arterial hypothermia may theoretically offer a superior risk-benefit profile compared to systemic hypothermia by enabling more direct and rapid cooling of the ischemic core and penumbra while minimizing systemic side effects.

Linking short-term (assessed by NIHSS) and long-term (assessed by mRS) outcomes, a pivotal question arises: does early improvement in NIHSS scores necessarily translate into enhanced long-term independent living capacity? Available data suggest that for systemic TTM combined with intravenous thrombolysis, the answer is likely negative; the modest or unstable improvement in short-term neurological signs failed to translate into clinically meaningful long-term functional benefits. For post-thrombectomy regional hypothermia, although a logical connection exists between the trend of improved short-term neurological scores (Wu et al., 2018) and the potential for improved long-term functional outcomes (Yue et al., 2023), larger-scale and rigorously designed clinical trials are still required to confirm this causal relationship and define the optimal treatment protocol (matching patient characteristics, intervention type, and timing). Future research should focus on identifying patient subpopulations most likely to benefit from specific TTM strategies through pre-specified subgroup analyses, and on elucidating the differential long-term impacts of various cooling modalities on neural repair pathways.

### 5.3 Adverse events associated with TTM

Although TTM demonstrates neuroprotective potential in the treatment of AIS, potential risk of adverse events warrants significant attention. Multiple clinical studies (Bi et al., 2011; Lyden et al., 2005; Thomas et al., 2010; Bart van der Worp et al., 2019; Horn et al., 2014; Chen et al., 2016; Wu et al., 2018; Yue et al., 2023; Bai et al., 2024; Krieger et al., 2001; Schwab et al., 2001; Martin-Schild et al., 2009; Su et al., 2016) have identified several adverse events, with reported incidence rates showing considerable variation. A detailed summary was provided in Table 2.

**TABLE 2:** Commonly reported adverse events and their incidence.

Category of adverse event	Specific manifestations	Incidence rate of surface cooling (%)	Incidence rate of endovascular cooling (%)
Infection	Pneumonia, Systemic Infection	18.4–48	23.9–38.5
Cardiovascular System	Arrhythmias, Hypotension	5.5–62	2.6–43.7
Coagulation Dysfunction	Intracranial Hemorrhage, Deep Vein Thrombosis	8.2–27.7	11.5–35.6
Electrolyte Disturbances	Hypokalemia	10-22.2	5.1–60
Mortality	All-Cause/Related Mortality	10.2–15	7.0–21.4

Effective management of adverse event risks is central to evaluating the risk-benefit ratio of TTM and ensuring safe clinical practice. In early clinical trials, shivering was the most common adverse reaction associated with therapeutic hypothermia. With improvements in study design, proactive pharmacological interventions (the use of magnesium, dexmedetomidine and pethidine) have significantly reduced the incidence and severity of shivering, improving the safety profile.

Both surface cooling and endovascular cooling report notable incidences of infection (Table 2). This is primarily attributed to the frequent need for deeper sedation to manage poorly controlled shivering during surface or endovascular cooling, which increases the risk of ventilator-associated pneumonia. Additionally, endovascular cooling carries the specific risk of catheter-related bloodstream infection. Compared to selective endovascular cooling, this adverse event appears more prominent in systemic endovascular cooling. Therefore, when selecting a cooling technique for patients with pre-existing pulmonary conditions or those anticipated to require prolonged sedation, the potential advantage of endovascular cooling in potentially reducing the need for deep sedation should be weighed. For patients with severely compromised immune function or colonization by drug-resistant bacteria, strict adherence to aseptic technique is essential. Consideration should also be given to pre-cooling infection screening, although the necessity of prophylactic antibiotic use remains controversial (Kallmünzer and Kollmar, 2011).

There is currently no evidence suggesting that the cardiovascular effects of hypothermia lead to adverse patient outcomes. However, severe shivering during hypothermic treatment significantly increases myocardial oxygen consumption and metabolic load, placing substantial stress on the cardiovascular system. This serves as a key driver for inducing tachyarrhythmias and blood pressure fluctuations, posing a considerable burden on patients with pre-existing cardiac dysfunction. To mitigate such adverse events, early identification of high-risk patients before initiating hypothermia is crucial. High-risk factors include advanced age, underlying cardiac conditions (coronary artery disease, heart failure and conduction blocks), electrolyte imbalances and hypovolemia (Adler et al., 2020; Kumar et al., 2022; Kirkegaard et al., 2022). Baseline assessments should include electrocardiography (ECG), echocardiography and electrolyte panels. Endovascular cooling is recommended for precise temperature control to minimize fluctuations, with preparatory measures such as atropine or defibrillators on standby if necessary.

Although previous experimental studies reported that hypothermia reduces platelet aggregation and prolongs coagulation time, clinically significant bleeding events causing hypothermia have not been observed so far. For patients with severe coagulation disorders, extremely low platelet counts, or high risk of active bleeding, surface cooling should be prioritized to avoid additional trauma and thrombosis risks associated with intravascular catheters. Regular monitoring of laboratory parameters, including platelet count, Prothrombin time/activated partial thromboplastin time (PT/APTT), fibrinogen, and D-dimer,- is essential. Bleeding at puncture sites, gingival bleeding, or melena should not be ignored. For patients undergoing endovascular cooling, daily examination of the punctured limb for swelling, skin temperature, color, pain, and circumference is necessary. Monitoring for new-onset symptoms such as dyspnea is also critical to prevent potential pulmonary embolism.

The core mechanism of electrolyte disturbances lies in the intracellular shift of ions induced by hypothermia. Compared to surface cooling, endovascular cooling exerts a more acute and pronounced effect on electrolyte balance, leading to a rapid intracellular translocation of electrolytes within a short timeframe, which can trigger arrhythmias and other complications. However, selective endovascular hypothermia appears to maintain more stable hemodynamic parameters and reduce metabolic disturbances. Therefore, electrolyte levels should be monitored before, during, and after rewarming during hypothermic therapy. Although hypothermic therapy has been associated with increased mortality risks, comparative analyses with control groups show no statistically significant differences in incidence rates. Furthermore, a direct causal relationship between patient mortality and adverse events resulting from hypothermic therapy is still unclear.

## 6 Conclusion

If applied in a timely and effective manner, TTM may be beneficial for alleviating both short- and long-term neurological damage in patients with AIS and promoting their prognosis. Although the advantages of TTM for AIS have become increasingly evident in recent years, most studies have been single-center studies with small sample sizes. In the future, it will be necessary to conduct multicenter large-scale clinical trials to validate the effects of TTM in patients with AIS. Furthermore, the optimal treatment protocol for TTM should be clarified in the clinical practice.
